# Association Between General Practitioner’s Sex and Documented Diagnoses of Female-Specific Disorders in German Primary Care: A Large Matched Cross-Sectional Study

**DOI:** 10.3390/clinpract16080152

**Published:** 2026-08-20

**Authors:** Karel Kostev, Ira Rodemer, Marcel Konrad, Matthias Kalder

**Affiliations:** 1 Department of Gynecology and Obstetrics, University Hospital, Marburg University, 35043 Marburg, Germany; 2 Epidemiology, IQVIA, 60486 Frankfurt am Main, Germany; 3 Health & Social, FOM University of Applied Sciences for Economics and Management, 60486 Frankfurt am Main, Germany

**Keywords:** general practice, physician sex, primary care, female-specific disorders, Germany

## Abstract

Background/Objectives: In Germany, women have direct access to gynaecologists without GP referral, making the GP’s role in female-specific conditions largely secondary. It has not previously been examined whether the sex of the GP is nonetheless associated with the documented prevalence of these conditions in primary care records. Methods: This retrospective cross-sectional study used data from the IQVIA™ Disease Analyzer. All women aged ≥18 years with at least one visit to one of 1183 GPs in Germany in 2025 were included. Women attending female and male GPs were matched 1:1 by propensity score on age, statutory health insurance, practice size, consultation frequency in 2025, and the van Walraven comorbidity score (excluding malignancy), yielding 661,485 women per group. Associations between GP sex and the documented prevalence of eight female-specific conditions were examined using conditional logistic regression within the age strata clinically relevant to each condition (from 18–30 to >75 years), with the age × GP-sex interaction tested in a single model per condition and E-values calculated to assess robustness to unmeasured confounding. Results: The association between GP sex and documented prevalence was strongly age-dependent, with a significant age × GP-sex interaction for seven of eight conditions (all *p* < 0.001). Female GP sex was associated with higher documented prevalence in mid-life, including menopausal and perimenopausal disorders (46–60 years: OR 1.37, 95% CI 1.28–1.46), endometriosis (31–45 years: OR 1.15, 1.03–1.28) and benign neoplasm of the female breast and genital organs (46–60 years: OR 1.16, 1.04–1.29); in the oldest women, the pattern attenuated or reversed (menopausal disorders > 75 years: OR 0.81, 0.72–0.90). No association was observed for breast cancer or female genital organ cancer at any age. Conclusions: In German primary care, the association between GP sex and the documented prevalence of female-specific disorders is age-dependent—most pronounced for conditions that require active clinical recognition and patient disclosure in mid-life, and absent for specialist-managed malignancies.

## 1. Introduction

In Germany, the healthcare of women with gynaecological and reproductive conditions is structured differently from many other countries. Unlike health systems in which the general practitioner (GP) acts as the gatekeeper to all specialist care, German women have direct and unrestricted access to gynaecologists without a GP referral. Gynaecologists are the primary responsible physicians for conditions such as endometriosis, menstrual cycle disorders, ovarian dysfunction, menopausal complaints, and gynaecological malignancies, and, because German women access them directly without GP referral, serve as the principal point of contact for these conditions for most women [1]. From adolescence onward, German women routinely establish an ongoing relationship with a gynaecologist, who takes primary responsibility for reproductive health monitoring, contraception, pregnancy care, and the management of female-specific disorders [1].

Within this system, the GP’s role in female-specific conditions is therefore largely secondary and not obligatory. GPs may encounter and document gynaecological diagnoses when patients present with relevant symptoms in the primary care setting, when specialist findings are communicated back to the GP practice, or when the GP takes on a coordinating function in complex or multimorbid patients. However, GPs are neither required nor routinely expected to conduct gynaecological examinations or to independently diagnose conditions such as endometriosis, ovarian dysfunction, or uterine pathology.

This structural feature of the German healthcare system raises an important and largely unexplored empirical question: if GPs are not the primary responsible physicians for female-specific disorders, do differences between male and female GPs nonetheless translate into measurable differences in how frequently these conditions are documented in the primary care record? Several mechanisms might produce such an association even in this context. Female patients may be more inclined to disclose gynaecological symptoms to a female GP, and female GPs may be more attentive to or proactive in recording such diagnoses when communicated by specialists [2,3]. Conversely, if documentation of these conditions in GP records is largely a passive reflection of specialist correspondence, GP sex may have little or no influence on recorded prevalence, and the majority of associations may be null—a finding that would itself be informative.

Physician sex is known to influence consultation behaviour, communication style, the delivery of preventive care, and the thoroughness of physical examination across a wide range of clinical domains [3,4,5]. A growing literature also documents that patients—particularly women—prefer to consult female physicians for conditions involving intimate examinations or reproductive health concerns, and report greater willingness to disclose sensitive symptoms when seen by a female physician [6]. It has not previously been examined whether these preferences and physician-level behavioural differences translate into measurable variation in the documented prevalence of female-specific conditions in routine primary care data.

Female-specific conditions offer an important test of this question across a wide clinical spectrum: they range from conditions almost entirely managed by gynaecologists (e.g., pregnancy, endometriosis, and gynaecological cancers) to those where GP involvement in recognition and documentation may be more variable (e.g., menopausal disorders, menstrual cycle complaints, and mastitis).

The aim of this study is to examine the association between GP sex and the documented prevalence of a broad range of female-specific conditions in a large representative German primary care population, including gynaecological disorders, benign neoplasms, reproductive conditions, and female cancers.

## 2. Materials and Methods

### 2.1. Data Source

This study drew on data from the IQVIA™ Disease Analyzer (Germany), a routinely collected, anonymized database covering diagnoses, prescriptions, and patient demographics from general and specialist practices throughout Germany. The representativeness of this database with respect to major chronic conditions and key patient characteristics in the German primary care context has been established previously [7]. Practice management systems transmit data on a monthly basis via encrypted, standardised interfaces, thereby retaining the structure of original clinical records. The database encompasses approximately ten million patients from around 2500 practices spanning eleven specialist fields, with sampling stratified by specialist group, federal state, community size, and physician age in accordance with annual statistics from the German Medical Association. Prior epidemiological research on female-specific conditions in Germany has drawn on this data source [8,9].

### 2.2. Study Design and Population

**Figure 1:**
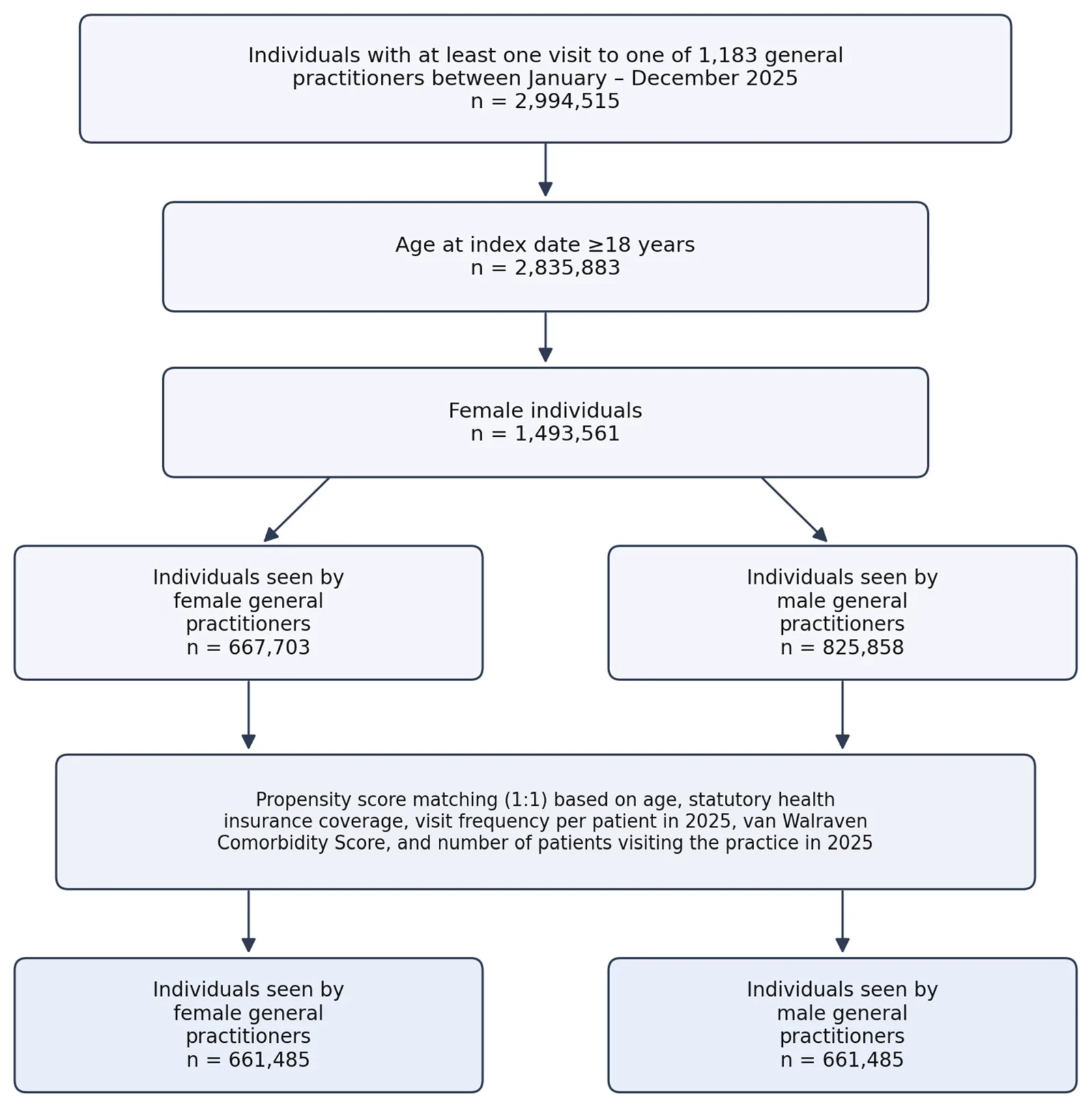
Selection of study population.

This retrospective cross-sectional study included all women with at least one visit to one of 1183 GPs in 2025. GPs were classified by sex (female vs. male), and the patient populations they served were examined accordingly. To achieve comparability between patient groups, women attending female and male GPs were matched 1:1 using nearest-neighbour propensity score matching. To address potential detection bias and residual confounding, the matching variables comprised patient age in years, type of health insurance (statutory vs. private), the total number of patients seen per physician in 2025, the number of consultations per patient in 2025 (a proxy for documentation opportunity), and the van Walraven comorbidity score. Because breast and gynaecological cancers were among the study outcomes, the malignancy categories were excluded from the comorbidity score to avoid overadjustment. The adequacy of covariate balance was evaluated using standardized mean differences, with values below 0.1 taken as evidence of satisfactory balance [10]. Matching produced 661,485 women per group; this equal group size is a direct consequence of the 1:1 matching design, in which each woman attending a female GP is paired with exactly one woman attending a male GP. The process of patient selection is illustrated in Figure 1.

### 2.3. Study Outcomes and Statistical Analysis

The primary outcome was the association between GP sex and the probability of a documented diagnosis of female-specific disorders. For each disorder, prevalence was calculated as the proportion of women with a confirmed or status-post diagnosis in 2025 among all women seen by the respective GPs in that year, expressed per 1000 patients.

For each condition, prevalence and the association with GP sex were estimated within the age strata that are clinically relevant for that condition, drawn from five bands (18–30, 31–45, 46–60, 61–75 and >75 years); biologically implausible strata—for example pregnancy-related codes beyond 45 years or menstrual-cycle disorders beyond 60 years—were not reported. This age-stratified approach was adopted because the conditions examined have markedly different age distributions and pooling across all ages would dilute clinically meaningful estimates. Within each stratum, a conditional logistic regression on the matched sample was fitted with the documented diagnosis as the dependent variable and GP sex (female vs. male) as the independent variable. The selection of conditions was guided by three criteria. First, conditions were restricted to those that are biologically exclusive to women or predominantly affect women, ensuring that any observed associations with GP sex could not be attributed to differences in the underlying sex composition of patient panels. Second, conditions were selected to represent a clinically meaningful spectrum of GP involvement: from conditions almost exclusively diagnosed and managed in specialist gynaecological practice (e.g., endometriosis, gynaecological cancers) to those where the GP may play a more active role in symptom recognition and documentation (e.g., menopausal disorders, mastitis). This range was chosen deliberately to test whether associations with GP sex differ according to the degree of primary care involvement. Third, conditions were required to be coded with dedicated ICD-10 codes routinely used in German primary care data and to have sufficient prevalence for stable regression estimates. Conditions examined were endometriosis (ICD-10: N80), benign neoplasm of female breast and genital organs (ICD-10: D24–D28), mastitis (ICD-10: N61), menopausal and other perimenopausal disorders (ICD-10: N95), menstrual cycle disorders (ICD-10: N91–N94), pregnancy, childbirth and the puerperium (ICD-10: O00–O99, Z32–Z39), breast cancer (ICD-10: C50), and female genital organ cancer (ICD-10: C51–C57).

Results are reported as odds ratios (ORs) with 95% confidence intervals (CIs) and *p*-values. To test whether the association with GP sex varied by age (effect modification), a single conditional logistic regression per condition was fitted across all ages, including GP sex and a GP sex × age interaction term (age modelled continuously); the *p*-value for this interaction term is reported. To assess robustness to unmeasured confounding, E-values were calculated for each statistically significant association, for both the point estimate and the confidence limit closest to the null. Given the exploratory, age-stratified nature of the analysis, emphasis was placed on the magnitude and cross-stratum consistency of the associations rather than on a single significance threshold. All analyses were performed using SAS version 9.4 (SAS Institute, Cary, NC, USA).

## 3. Results

The selection of the study population is shown in Figure 1. After 1:1 propensity score matching, the study sample comprised 661,485 women attending female GPs and 661,485 women attending male GPs, for a total of 1,322,970 individuals.

Baseline characteristics of the matched sample are presented in Table 1. The mean age was 54.3 years (SD 20.0) among women attending female GPs and 54.4 years (SD 20.1) among those attending male GPs (SMD 0.001). The groups were closely balanced with respect to statutory health insurance (93.2% vs. 92.8%; SMD 0.015), the number of patients seen per physician in 2025 (median 1380 vs. 1324; SMD 0.004), consultation frequency (median 3 vs. 3 consultations per patient; SMD 0.010) and the van Walraven comorbidity score (mean 0.92 vs. 0.93; SMD 0.003). All standardized mean differences were well below 0.1, indicating adequate covariate balance following matching.

The associations between GP sex and the documented prevalence of female-specific disorders, stratified by clinically relevant age groups, are shown in Table 2. The association was strongly age-dependent: the age × GP-sex interaction was statistically significant for seven of the eight conditions (all *p* < 0.001), the exception being benign neoplasm of the female breast and genital organs (*p* = 0.137). Female GP sex was associated with a higher documented prevalence of menopausal and perimenopausal disorders in mid-life, most markedly at 46–60 years (17.84 vs. 12.98 per 1000; OR 1.37, 95% CI 1.28–1.46) and at 31–45 years (OR 1.32, 1.09–1.60), whereas in women older than 75 years, the direction reversed (OR 0.81, 0.72–0.90). Endometriosis was more frequently documented by female GPs at 31–45 years (OR 1.15, 1.03–1.28), as was benign neoplasm of the female breast and genital organs at 46–60 years (OR 1.16, 1.04–1.29). For pregnancy, childbirth and the puerperium, a modest positive association was present at 31–45 years (OR 1.08, 1.02–1.14). Menstrual cycle disorders were less frequently documented by female GPs in the youngest women (18–30 years: OR 0.90, 0.85–0.95). No association with GP sex was observed for breast cancer or female genital organ cancer in any age stratum. E-values for the statistically significant associations (Table 3) ranged from 1.36 to 2.08 for the point estimates and from 1.16 to 1.89 for the confidence limits closest to the null, indicating that an unmeasured confounder would need a moderately strong association with both GP sex and the outcome to fully explain the observed associations.

## 4. Discussion

This large matched cross-sectional study examined the association between GP sex and the documented prevalence of eight female-specific conditions in German primary care and, in contrast to an unstratified analysis, showed that this association is strongly age-dependent. A statistically significant age × GP-sex interaction was present for seven of the eight conditions. Female GP sex was associated with higher documented prevalence of hormone- and reproduction-related conditions—menopausal and perimenopausal disorders, endometriosis, and benign neoplasms of the breast and genital organs—predominantly in mid-life (roughly 31–60 years), whereas no association was seen for breast or female genital organ cancer at any age.

The age-stratified analysis materially refines the interpretation of these findings. For menopausal and perimenopausal disorders, the association with female GP sex was concentrated in the peri- and early post-menopausal window (31–60 years), where non-specific symptoms most require active clinical attribution, and it reversed in women older than 75 years. This is consistent with the hypothesis that female GPs more readily recognise and document menopause-related symptoms when they are clinically relevant, rather than with a true difference in underlying prevalence. To keep the estimates clinically interpretable, each condition is reported only within the age range in which it is biologically plausible. The absence of any association for gynaecological cancers across all age strata reinforces the view that, where the primary care record functions largely as a passive repository of specialist-generated diagnoses, GP sex has little influence on what is documented.

The strongest association observed was for menopausal and perimenopausal disorders, which were recorded approximately 23% more often in patients of female GPs. These significant findings may be justified by the fact that female GPs are more likely than their male colleagues to classify nonspecific symptoms, such as hot flashes, sleep disturbances, mood swings, or urogenital symptoms, in women as part of the spectrum of menopausal and perimenopausal conditions. This is supported by evidence that female physicians deliver female-specific preventive procedures more frequently than their male counterparts [5,11], and that they conduct longer consultations, ask more questions, and elicit more detailed clinical information from patients [4]. At the same time, women themselves report a greater willingness to disclose sensitive or intimate health concerns to female physicians and are more likely to raise gynaecological symptoms in a gender-concordant consultation [12,13]. Menopausal disorders occupy a particular position in this context: they are by their nature age-related and may present with non-specific symptoms—including mood changes, sleep disturbances, and fatigue—that require active clinical attribution to be coded as a menopausal disorder in the GP record. Evidence from electronic health record studies indicates that documented menopause diagnoses have risen in recent years [14] and that menopausal symptoms, although common in mid-life, are captured predominantly in specialist rather than primary care records, with only a small proportion documented by family physicians [15], suggesting that the higher documentation rates in practices of female GPs may reflect greater clinical attention to this condition rather than a true difference in prevalence. The observation that menopausal disorders showed the strongest association in the present study may therefore reflect a combination of patients being more willing to report symptoms and physicians being more inclined to recognise and document them.

The associations observed for endometriosis and benign neoplasms are notable given that both conditions are predominantly diagnosed and managed in secondary care in Germany. In a healthcare system where women access gynaecologists directly and without referral [1], GP documentation of such diagnoses is to a substantial degree a reflection of whether specialist findings are passed back to and recorded by the GP. The structural analysis of GP–gynaecologist collaboration in Germany has shown that while around 38% of gynaecologists routinely send clinical notes to GPs, only a small minority of GP practices have formalised agreements for mutual referral or data exchange [16]. Against this backdrop, the observed association between female GP sex and higher documentation of endometriosis and benign neoplasms may reflect systematic differences in how actively female GPs incorporate specialist correspondence into the primary care record, or in how readily patients volunteer this information during the GP consultation.

On the other hand, the hypothesis discussed in relation to menopausal symptoms could also apply here. Female GPs tend to attribute nonspecific symptoms in the lower abdomen or breasts more frequently to gynaecological conditions, such as endometriosis or benign tumors in the lower abdomen. The same appears to apply to the diagnosis of mastitis.

The null findings for gynaecological cancer diagnoses and for conditions closely linked to ongoing specialist management—such as female genital organ cancer and breast cancer—are consistent with the hypothesis that GP sex has little influence on documentation when the primary care record functions largely as a passive repository of specialist-generated diagnoses. In such cases, the frequency of documentation depends more on the regularity of specialist correspondence than on the GP’s own clinical behaviour or communication style. A systematic review on primary care clinicians’ interactions with women with gynaecological conditions found that documentation and recognition are impeded less by physician-level factors than by structural constraints, including fragmented care pathways and limited integration of specialist findings into primary care records [17]. The null findings in the present study for cancer diagnoses align with this interpretation.

The finding that female GP sex was associated with a lower documented prevalence of menstrual cycle disorders only in the youngest women (18–30 years), with no association at older ages, is noteworthy, as these conditions involve symptoms that require patient disclosure and may be subject to the same communication dynamics discussed above. One possible explanation is that menstrual disorders are commonly raised in the context of gynaecological consultations in Germany and may not routinely reach the GP record at all, irrespective of GP sex. Furthermore, this primarily affects younger women of reproductive age, often in connection with their fertility or contraception, topics they seem to discuss solely with their gynaecologist.

Alternatively, the prevalence of these conditions in GP records may reflect referral patterns or prescription triggers that are independent of the GP’s sex. The study by Boulis and Long [18], which found that physician gender effects on clinical practice were most pronounced for genital-specific conditions rather than broadly gynaecological ones, provides some support for the heterogeneity of effects observed here.

The present study has several strengths. The large, matched sample reduces confounding by the matched characteristics (patient age, insurance status, and practice size), and the use of routinely collected primary care data supports the external validity of the findings (i.e., the degree to which they reflect real-world clinical practice rather than a selected or experimental setting). Associations were interpreted with emphasis on their magnitude and consistency across adjacent age strata and supported by E-values, rather than on a single significance threshold. Nevertheless, a number of limitations should be noted. First, the cross-sectional design does not allow causal conclusions to be drawn; the observed associations may reflect differences in documentation behaviour, patient self-selection, consultation patterns, or a combination of these factors. Moreover, these documentation and coding differences could not be externally validated against clinical records. Second, the database does not capture variables such as physician workload characteristics beyond practice size, or the frequency and content of specialist correspondence, all of which could confound or mediate the observed associations. Third, the study period covers a single calendar year (2025), and it is unclear whether the observed patterns reflect stable structural differences or year-specific variation. Fourth, the analysis was restricted to German primary care, and the findings may not be transferable to health systems with different models of GP–specialist interaction. Fifth, because the study population was restricted to women, patient–physician gender concordance could not be separated from an effect of GP sex per se; establishing whether concordance itself drives the observed differences would require a design that also includes male patients. Sixth, the age-stratified design entails multiple comparisons, which increases the risk of chance findings; we therefore emphasised associations that were consistent across adjacent strata and supported by E-values, as described above. Finally, the comorbidity adjustment relied on a modified van Walraven score from which malignancy categories were removed, so residual confounding by cancer-related comorbidity cannot be entirely excluded.

These findings have practical implications for primary care. The age-dependent pattern indicates that differences between male and female GPs in documenting female-specific conditions are concentrated in symptom-driven, actively recognised conditions during the reproductive and menopausal years, rather than in specialist-managed disease. This suggests that measures aimed at reducing gender-related variation in women’s health documentation—for example, structured symptom checklists for menopausal and gynaecological complaints, and clearer pathways for incorporating specialist correspondence into the primary care record—are likely to be most useful for mid-life women, and that improving GP–gynaecologist communication may reduce documentation gaps independently of physician sex.

Future research should investigate whether the associations identified here are explained by differences in patient disclosure, physician recording practice, or differences in the integration of specialist information into primary care records, and whether comparable patterns are observed in other health systems.

## 5. Conclusions

In conclusion, this study found that the association between GP sex and the documented prevalence of female-specific conditions in German primary care is age-dependent. Female GP sex was associated with higher documented prevalence of menopausal and perimenopausal disorders, endometriosis and benign neoplasms of the female breast and genital organs, predominantly in mid-life, while no association was observed for gynaecological cancers at any age and the association attenuated or reversed in the oldest women. These findings support an influence of GP sex on the documentation of female-specific disorders that is largely confined to conditions in which active clinical recognition and patient disclosure play a role, and not to conditions whose documentation depends primarily on specialist correspondence.

These findings support the association between the gender of the primary care physician and documentation practices for conditions in which a visit to the primary care physician provides a useful opportunity to interpret nonspecific symptoms within the clinical context of various medical specialties. However, this does not apply to conditions for which documentation in the primary care record depends largely on correspondence with specialists.

## Figures and Tables

**Table 1:** Baseline characteristics of the study sample after 1:1 matching.

Variable	Individuals Seen by Female GPs (*n* = 661,485)	Individuals Seen by Male GPs (*n* = 661,485)	SMD
Mean age, years (SD)	54.3 (20.0)	54.4 (20.1)	0.001
Age 18–30 years, *n* (%)	98,856 (14.9)	100,765 (15.2)	
Age 31–45 years, *n* (%)	140,351 (21.2)	138,752 (21.0)	
Age 46–60 years, *n* (%)	154,686 (23.4)	154,064 (23.3)	
Age 61–75 years, *n* (%)	155,294 (23.5)	151,935 (23.0)	
Age > 75 years, *n* (%)	112,298 (17.0)	115,969 (17.5)	
Statutory health insurance, *n* (%)	616,309 (93.2)	613,802 (92.8)	0.015
Private health insurance, *n* (%)	45,176 (6.8)	47,683 (7.2)	
Patients seen per physician in 2025, median (IQR)	1380 (933)	1324 (946)	0.004
Consultations per patient in 2025, median (IQR)	3 (5)	3 (4)	0.010
van Walraven comorbidity score (excl. malignancy), mean (SD)	0.92 (3.29)	0.93 (3.26)	0.003

Data are *n* (%) unless otherwise specified. SMD, standardized mean difference; IQR, interquartile range; SD, standard deviation; GP, general practitioner. The van Walraven comorbidity score excludes malignancy categories, as breast and gynaecological cancers were study outcomes. All SMDs were <0.1, indicating adequate covariate balance after matching.

**Table 2:** Age-stratified association between general practitioner sex and the documented prevalence of female-specific disorders.

Condition (ICD-10)	Age Group, Years	Prevalence per 1000, Female GPs	Prevalence per 1000, Male GPs	OR (95% CI)	*p*-Value	*p* for Interaction (Age × GP Sex)
Endometriosis (N80)	18–30	4.82	4.98	1.02 (0.88–1.18)	0.828	<0.001
	31–45	6.12	5.59	1.15 (1.03–1.28)	0.014	
	46–60	2.11	2.07	1.06 (0.89–1.25)	0.523	
Mastitis (N61)	18–30	0.81	0.61	1.39 (0.94–2.05)	0.096	<0.001
	31–45	1.10	0.88	1.21 (0.93–1.58)	0.154	
	46–60	0.50	0.49	0.92 (0.65–1.31)	0.638	
Menopausal and other perimenopausal disorders (N95)	31–45	2.08	1.61	1.32 (1.09–1.60)	0.005	<0.001
	46–60	17.84	12.98	1.37 (1.28–1.46)	<0.001	
	61–75	8.47	8.53	0.99 (0.91–1.08)	0.884	
	>75	7.12	8.55	0.81 (0.72–0.90)	<0.001	
Menstrual cycle disorders (N91–N94)	18–30	32.20	36.38	0.90 (0.85–0.95)	<0.001	<0.001
	31–45	14.60	13.98	1.03 (0.96–1.10)	0.465	
	46–60	6.46	6.09	1.05 (0.95–1.17)	0.296	
Pregnancy, childbirth and the puerperium (O00–O99, Z32–Z39)	18–30	21.67	22.14	0.98 (0.91–1.05)	0.579	<0.001
	31–45	24.80	23.29	1.08 (1.02–1.14)	0.008	
Benign neoplasm of female breast and genital organs (D24–D28)	18–30	0.75	0.72	1.12 (0.75–1.67)	0.582	0.137
	31–45	2.99	2.82	1.08 (0.92–1.26)	0.357	
	46–60	5.17	4.79	1.16 (1.04–1.29)	0.010	
	61–75	3.00	2.86	1.04 (0.90–1.20)	0.617	
	>75	1.59	1.88	0.88 (0.70–1.11)	0.282	
Breast cancer (C50)	18–30	0.84	0.69	1.08 (0.74–1.58)	0.696	<0.001
	31–45	3.15	3.37	0.87 (0.75–1.00)	0.059	
	46–60	10.82	11.61	0.94 (0.87–1.01)	0.100	
	61–75	17.81	17.97	1.00 (0.94–1.06)	0.923	
	>75	22.73	21.30	1.06 (0.99–1.13)	0.074	
Female genital organ cancer (C51–C57)	18–30	0.22	0.27	1.01 (0.52–1.95)	0.978	<0.001
	31–45	1.28	1.27	1.03 (0.81–1.31)	0.797	
	46–60	2.95	3.19	0.92 (0.80–1.06)	0.236	
	61–75	4.57	4.90	0.92 (0.82–1.03)	0.153	
	>75	5.27	4.83	1.04 (0.91–1.19)	0.542	

Prevalence is expressed per 1000 women within each age band. Only age strata that are clinically relevant for each condition are shown. OR, odds ratio (reference = male GP) from conditional logistic regression on the matched sample within each age stratum; CI, confidence interval; GP, general practitioner. *p* for interaction is the Wald test of the age × GP-sex product term (patient age modelled continuously) from a single model per condition, fitted across the full modelled age range. ICD-10 codes are shown in parentheses.

**Table 3:** Sensitivity analysis: E-values for the statistically significant (*p* < 0.05) age-stratified associations between general practitioner sex and documented female-specific disorders.

Condition (ICD-10)	Age Group, Years	OR (95% CI)	E-Value (Point Estimate)	E-Value (CI Limit Closest to Null)
Endometriosis (N80)	31–45	1.15 (1.03–1.28)	1.56	1.19
Menopausal and other perimenopausal disorders (N95)	31–45	1.32 (1.09–1.60)	1.97	1.39
Menopausal and other perimenopausal disorders (N95)	46–60	1.37 (1.28–1.46)	2.08	1.89
Menopausal and other perimenopausal disorders (N95)	>75	0.81 (0.72–0.90)	1.78	1.46
Menstrual cycle disorders (N91–N94)	18–30	0.90 (0.85–0.95)	1.48	1.29
Pregnancy, childbirth and the puerperium (O00–O99, Z32–Z39)	31–45	1.08 (1.02–1.14)	1.36	1.16
Benign neoplasm of female breast and genital organs (D24–D28)	46–60	1.16 (1.04–1.29)	1.59	1.23

Only age strata with a statistically significant association (*p* < 0.05) are shown. The E-value is the minimum strength of association, on the risk-ratio scale, that an unmeasured confounder would need with both GP sex and the outcome to explain away the observed association; because outcomes are rare, the odds ratio approximates the risk ratio. The point-estimate E-value refers to the OR; the CI E-value refers to the confidence limit closest to the null. OR, odds ratio; CI, confidence interval; GP, general practitioner.

## Data Availability

The datasets presented in this article are not readily available because of strict privacy and confidentiality requirements; legal restrictions prohibit sharing raw data with third parties.
